# Antiviral Response in Pandemic Influenza Viruses

**DOI:** 10.3201/eid1201.051186

**Published:** 2006-01

**Authors:** Adolfo García-Sastre

**Affiliations:** * Mount Sinai School of Medicine, New York, New York, USA

**Keywords:** influenza, interferon, NS1, perspective

## Abstract

The outcome of viral infections depends on a complex set of interactions between the viruses and their hosts. Particularly, viral infection triggers specific signaling programs within the infected cells that results in substantial changes in host gene expression. While some of these changes might be beneficial for viral replication, others represent the induction of a host antiviral response. In this respect, viruses have evolved genes that counteract this initial innate antiviral response. These viral-host interactions shape the subsequent phases of the disease and influence the adaptive immune response. In influenza viruses, the nonstructural protein 1 inhibits the interferon-mediated antiviral response. The regulatory activities of this viral protein play a major role in the pathogenicity of influenza virus and appear partially responsible for the ability of influenza viruses to infect multiple animal species, which likely contributes to the generation of new pandemic viruses in humans.

## Abstract 2

The regulatory activities of the nonstructural protein 1 appear to affect the ability of influenza viruses to infect multiple animal species.

Coevolution of pathogens with their hosts has resulted in the shaping of the host immune system. A major component of this system is the innate immune response, which includes all the host barriers and responses with broad specificity against pathogens. The innate immune response not only represents the first barrier against infection but also provides the appropriate signals required for the subsequent adaptive cellular and humoral immune responses to develop. The type I interferon (IFNα/β) response constitutes a critical element of the innate immune system that is particularly important in the battle against viral pathogens. Secretion of IFNα/β results in the induction of a cellular antiviral response involving the transcriptional upregulation of >100 genes (*1*).

Despite the host's sophisticated immune system, viruses continue to successfully infect them and cause disease and, in some cases, death. The success of viruses is explained, at least in part, by the acquisition of viral genes during evolution that antagonize the host immune response. Viral-encoded IFNα/β antagonists are of particular interest, since they appear to be present in most animal viruses. We detail how influenza viruses evade the host innate immunity, with particular emphasis on the IFNα/β response, and the implications of this immune evasion in pandemic influenza.

## IFNα/β Antiviral Response

Animal cells that sense viral infection respond almost immediately by synthesizing and secreting IFNα/β. The IFNα/β genes include IFNβ and many closely related IFNα genes. Most cells have intracellular sensors of viral products that, when activated, initiate a signaling cascade that results in transcriptional induction of the IFNβ gene. The nature of these sensors has remained unknown until recently, when 2 putative RNA helicases, RIG-I and MDA-5, were identified as sensors for viral dsRNA generated in the cytoplasm during viral infection (*2**–**4*). Binding to dsRNA by these proteins may result in initiation of helicase activity, concomitant with a conformational change that leads to recruiting additional cellular factors, including the recently identified IPS-1/MAVS protein (*5**,**6*). As a result, different cellular kinases, including the IRF3 kinases TBK1 and IKKε, become activated. Activated IRF3, together with NF-κB and AP-1, accumulate in the nucleus, bind to the IFNβ promoter, and stimulate transcription. While cytoplasmic viral dsRNA is one of the viral molecules that trigger this cascade, other viral products and other cellular sensor molecules also likely participate in the induction of IFNβ. IFNα/β induction is also stimulated by the presence of viral RNA and DNA in the endosome through the action of TLR3, TLR7, TLR8, or TLR9 (*7*). Different cell subtypes appear to employ different mechanisms to recognize viral products (*8*).

Once IFNα/β has been synthesized, it is secreted and binds to the IFNα/β receptor. All IFNαs and IFNβ bind to the same receptor and as a result, the cytoplasmic kinases JAK1 and TYK2 become activated and phosphorylate the STAT1 and STAT2 molecules. This process promotes generation of the ISGF3 transcription factor, a complex of STAT1, STAT2, and IRF9 that accumulates in the nucleus. Nuclear ISGF3 binds to promoters that contain interferon-stimulated response elements and stimulates the transcriptional induction of antiviral genes, including MxA, PKR, OAS, ADAR, PML, p56, and many others (*9*). These IFN-stimulated genes inhibit viral replication by many different mechanisms, including binding to viral nucleocapsids, translation inhibition, RNA degradation, RNA editing, and apoptosis induction. Moreover, secreted IFNα/β promotes the generation of robust cellular and humoral immunity (*10**,**11*). In general, the IFNα/β response has a complex regulation that involves positive and negative feed-back mechanisms, some of which are still unknown.

## Nonstructural Protein 1 of Influenza Virus

Although IFNα/β was first described as a factor with antiviral activity secreted by cells treated with partially heat-inactivated influenza A viruses (*12*), it was also recognized early on that influenza viruses are poor IFNα/β inducers (*13*). This is because influenza viruses, like many other viruses, encode mechanisms to evade and antagonize the IFNα/β response (*14*). In the case of influenza A virus, this IFNα/β antagonistic function is encoded by the nonstructural protein 1 (NS1) gene.

NS1 of influenza A viruses is encoded by the unspliced mRNA derived from the shortest RNA segment of the 8 viral RNA segments. The protein is the most abundant nonstructural viral protein expressed in influenza A virus–infected cells. The development of reverse genetics techniques to manipulate the influenza virus genome made it possible to generate NS1 mutant viruses, including a recombinant influenza A virus lacking the NS1 gene (*15*). The NS1 knockout influenza A virus, delNS1, was replication defective in most cells and hosts, except for those lacking a functional IFNα/β system. Most remarkable, delNS1 virus was highly attenuated in mice but replicated and caused disease in STAT1 knockout mice, which lack one of the key transactivator molecules needed for the IFNα/β response (*15*). These results indicate that NS1 is required to overcome the IFNα/β response during influenza A virus infection.

The basis of the IFNα/β antagonistic properties of the NS1 of influenza A virus relies on its ability to prevent IFNβ synthesis; this explains the poor IFNβ–inducing properties of influenza A viruses (*16**,**17*). In the absence of NS1, influenza A virus becomes a high IFNα/β–inducing virus, and induction of high levels of IFNα/β results in inhibition of replication of delNS1 virus. NS1, by virtue of its dsRNA binding properties, is likely to sequester viral dsRNA produced during viral infection, which prevents recognition of this dangerous molecule by cellular sensors. This model of action is consistent with the ability of NS1 expression to prevent activation of transcription factors involved in the induction of IFNα/β synthesis, including IRF3 (*16*). Moreover, dsRNA binding is required for optimal inhibition of IFNβ production by NS1 (*18*). Similar results were obtained with the NS1 of influenza B virus (*19**,**20*). However, interactions of NS1 with cellular proteins also likely contribute to its IFNα/β antagonistic functions (*21*). NS1 of influenza A virus, but not of influenza B virus, inhibits cellular factors involved in mRNA processing (*22**,**23*); this function might also play a role in inhibiting IFNα/β production by influenza A virus (*24*). Finally, NS1 has also been shown to have IFNα/β inhibitory properties at a post-IFNα/β synthesis level. The NS1 of both influenza A and B viruses prevents the activation of the translation inhibitory and IFN inducible protein PKR (*25**,**26*); the NS1 of influenza B virus inhibits the activity of ISG15 (*27*), an IFN-inducible protein that enhances the IFN-mediated antiviral response.

## Role of NS1 Gene

Influenza A viruses can infect many different animal species, such as different birds (e.g., waterfowl, chickens, turkeys), horses, pigs and humans, but also cross species, with avian strains infecting mammalian species, including humans. This property is especially critical during human pandemics that are characterized by novel antigenic determinants. These determinants derive from avian strains for which no immunity exists in most human population, which results in higher illness and death rates. The factors involved in the ability of an avian influenza virus strain, or of a reassortant virus containing avian antigenic determinants, to infect and propagate in humans are poorly understood; this lack of knowledge hampers our ability to predict the pandemic potential of avian influenza virus strains circulating in birds. Although the receptor specificity of the hemagglutinin protein is a factor that appears to be important for human adaptation of avian strains, other poorly understood factors also participate in this adaptation (*28*). With respect to NS1, viral strains from different animal hosts likely have NS1 genes adapted to antagonize the IFNα/β system of their specific host species. This was the case when the NS1 gene of the human influenza A virus that caused the 1918 H1N1 pandemic was compared with the NS1 gene of the mouse-adapted H1N1 influenza A virus strain WSN. Replacement of the NS1 gene of WSN virus with that of the 1918 virus resulted in an attenuated virus in mice, but this virus more efficiently inhibited the IFNα/β system in human cells (*21**,**29*). This inhibition might be explained by specific interactions of NS1 with host factors that have different sequences depending on the host, with NS1 of a mouse-adapted strain interacting better with murine factors than with human factors, and vice versa. If this is a general property of NS1 from different influenza virus strains, an avian strain would require adaptation of its NS1 gene to efficiently antagonize the human IFNα/β system. Alternatively, an avian strain would require acquisition by reassortment of an NS1 gene from a human strain to efficiently infect and propagate in humans.

Because mutations that affect NS1 function also have a profound effect on viral pathogenicity, highly pathogenic influenza virus strains may have an NS1 gene with particularly strong IFNα/β antagonistic properties. Moreover, the ability of NS1 to attenuate the activation of different transcription factors during viral infections has implications beyond the inhibition of IFNα/β synthesis. For instance, expression of many other cytokines and molecules involved in activation of dendritic cell function also appear to be regulated by NS1 (*30*). In this respect, the NS1 of the highly pathogenic avian H5N1 viruses circulating in poultry and waterfowl in Southeast Asia might be responsible for an enhanced proinflammatory cytokine response (especially TNFa) induced by these viruses in human macrophages (*31**,**32*). High levels of proinflammatory cytokines are likely to play an important role in the unusual lethality of these viruses in humans. Fortunately, infection with these viruses appears to be rare and the viruses have not been able to efficiently propagate from human to human.

## Other Influenza Antagonists of Host Response

Although delNS1 influenza virus is a high inducer of IFNα/β, partial UV inactivation of this virus results in even higher induction of IFNα/β (*33*). These results suggest the presence of additional viral genes besides NS1 that attenuate IFNα/β production during viral infection and that become inactivated by UV. The viral polymerase, possibly through its endonuclease "cap-snatching" activity, might be responsible for this anti- IFNα/β activity (*33*). Further experimentation will be required to evaluate this hypothesis. In any case, the presence of multiple viral genes that cooperatively antagonize the IFNα/β response is not uncommon among the different virus families.

Influenza A virus encodes a second nonstructural polypeptide in virus-infected cells, the PB1-F2 protein (*34*). This protein is encoded by an alternative open reading frame of the PB1 RNA segment, which also directs the synthesis of the PB1 protein, a critical component of the viral polymerase. The PB1-F2 protein localizes to the mitochondria of the infected cells (*35*) where it interacts with 2 components of the mitochondrial permeability transition pore complex, ANT3 and VDCA1, that are thought to play a major role in apoptosis control (*36*). As a result, expression of PB1-F2 sensitizes cells to apoptosis. This process might constitute an important immune evasion strategy. Thus, a PB1-F2-knockout influenza A virus induced less cell death than the wild-type virus in infected human monocytes, which suggests that expression of PB1-F2 affects immune cell function during viral infection (*34*). Although several influenza A virus strains that lack PB1-F2 occur naturally, PB1-F2 likely contributes to viral pathogenicity and might have an important role in determining the severity of pandemic influenza.

## NS1 as Target for Antivirals and Vaccines

Our knowledge of NS1 function might be applied in the near future to select for new antiviral compounds against influenza virus. Predictably, small molecules that interfere with the ability of NS1 to bind dsRNA or prevent IFNα/β production will also enhance the host innate immunity against influenza virus, resulting in faster viral clearance. In addition, recombinant influenza viruses with impaired NS1 function might represent efficient live attenuated vaccines against influenza. These viruses can be grown in IFNα/β-deficient substrates to high titers, but they are attenuated in the host (*37*). Moreover, since the inhibitory effects of NS1 attenuate aspects of both innate and adaptive immunity, NS1 mutant viruses appear to be intrinsically more immunogenic (*38*). Recombinant influenza viruses with modified NS1 genes have been developed and have proven to be attenuated and immunogenic in different animal models. These modified viruses might be used in the future as the basis of live vaccines against epidemic and pandemic influenza (*37**–**40*).
